# Linking avian communities and avian influenza ecology in southern Africa using epidemiological functional groups

**DOI:** 10.1186/1297-9716-43-73

**Published:** 2012-10-26

**Authors:** Alexandre Caron, Michel de Garine-Wichatitsky, Mduduzi Ndlovu, Graeme S Cumming

**Affiliations:** 1 Cirad, AGIRs, RP-PCP, Harare, Zimbabwe; 2 Cirad, AGIRs, Department ES, Montpellier, France; 3 MRI, Department of Entomology & Zoology, University of Pretoria, Pretoria, South Africa; 4 Percy FitzPatrick Institute, DST/NRF Centre of Excellence, University of Cape Town, Rondebosh, Cape Town, 7701, South Africa

## Abstract

The ecology of pathogens, and particularly their emergence in multi-host systems, is complex. New approaches are needed to reduce superficial complexities to a level that still allows scientists to analyse underlying and more fundamental processes. One promising approach for simplification is to use an epidemiological-function classification to describe ecological diversity in a way that relates directly to pathogen dynamics. In this article, we develop and apply the epidemiological functional group (EFG) concept to explore the relationships between wild bird communities and avian influenza virus (AIV) in three ecosystems in southern Africa. Using a two year dataset that combined bird counts and bimonthly sampling for AIV, we allocated each bird species to a set of EFGs that captured two overarching epidemiological functions: the capacity of species to maintain AIV in the system, and their potential to introduce the virus. Comparing AIV prevalence between EFGs suggested that the hypothesis that anseriforms (ducks) and charadriiforms (waders) drive AIV epidemiology cannot entirely explain the high prevalence observed in some EFGs. If anseriforms do play an important role in AIV dynamics in each of the three ecosystems, the role of other species in the local maintenance of AIV cannot be ruled out. The EFG concept thus helped us to identify gaps in knowledge and to highlight understudied bird groups that might play a role in AIV epidemiology. In general, the use of EFGs has potential for generating a range of valuable insights in epidemiology, just as functional group approaches have done in ecology.

## Introduction

The ecology of pathogens and the emergence of diseases in multi-host systems are complex [1,2]. Understanding epidemiology often requires the incorporation of a wide variety of different kinds of evidence and disciplinary approaches [3]. Traditional surveillance and control approaches have often focused on humans, domestic animals, and known vectors. However, an increasing body of information indicates that effective disease surveillance and control may be heavily dependent on understanding the epidemiology of pathogens in relation to the ecology of their wild hosts e.g., [4-7].

Avian Influenza Viruses (AIVs) in wild birds have recently received increased attention due to the emergence of the Highly Pathogenic AIV H5N1 strain and its potential threat to human health [8]. Although numerous studies of low pathogenic AIV strains (LPAI) in waterfowl and wild birds have been published, encompassing tens of thousands of sampled wild birds, we still know relatively little about the susceptibility of individual bird species to AIV in relation to the global number of bird species [7]. The avian community in a single ecosystem can include hundreds of interacting species. In addition, the response of bird species to specific AIV subtypes (16 hemagglutinins and 9 neuramidases known) is variable and prevalence patterns of specific subtypes will be determined by the bird cenosis. So far, most studies of AIV have concentrated on anseriforms and to a lesser extent on charadriiforms, which are known to be reservoirs for LPAI [7,9]. In their synthesis of wild bird low pathogenic avian influenza surveillance worldwide, Olsen et al. [7] found that out of more than 90 000 birds sampled, 54% were anseriforms and 25% charadriiforms. As a consequence, little information on AIV prevalence in the rest of the avian community has been published, and much of what has been published has been obtained as “by-catch” from capture protocols that have been focused on ducks. The minimum sample sizes that would be necessary to confidently estimate prevalence for most non-target bird species are often not reached, with the risk that the common practice of focusing mainly on anseriforms may be overlooking the role of other bird groups in the epidemiology of AIV in waterfowl communities.

Biases in the selection of species to sample are not the only problem in available data sets for AIV. The comparison between sample and community composition is a fundamental parameter in epidemiological studies [10]. In many cases, a lack of information regarding the composition of the wild bird community from which the sample is taken makes conclusions from AIV studies even harder to interpret. A total of 100 positive samples from mallard ducks (*Anas platyrhynchos*), for example, carries quite different epidemiological implications if mallards represent 0.1% versus 90% of the number of wild birds present in the ecosystem; and similarly, the relevance of 100 positive samples from mallards differs if the system contains 10 or 100 other species. Interpretation of the role of a species in pathogen maintenance cannot be done rigorously without considering the potential role of the rest of the community. The sampling bias that is attendant on any field captures of wild birds should therefore be a crucial parameter in wildlife epidemiological studies.

As more host species are considered in a host-pathogen system, the number of potential interactions (and hence, the complexity of potential pathogen transmission pathways) increases exponentially in relation to the number of species in the analysis. The problem is further complicated, in the case of AIV, by the existence of a diversity of viral subtypes and substantive variations in pathogen-host interactions (e.g. susceptibility and pathogenicity). It rapidly becomes both empirically and computationally unfeasible to analyse the specific relationships between each host species and each pathogen subtype; and assigning each host species to a specific role in the epidemiological cycle (e.g. reservoir, dead end-host, spreader) can be extremely difficult even when large, detailed data sets are available. Often, in such contexts, management and policy responses must nonetheless occur. The control of emerging pathogens, for instance, typically requires rapid responses that are based on partial and imperfect information. There is therefore a clear need for techniques that can be used to summarize epidemiological complexity without oversimplifying it, even if resulting conclusions are later modified by the findings of more intensive studies.

This kind of problem, in which the number of interacting elements and interactions rapidly exceeds what can reasonably be measured in a typical scientific study, is common in research on complex systems. For example, overwhelming complexity generated by multiple interacting influences is a unifying problem in research on systems as diverse as gene expression, stock markets, and ecosystems. Epidemiologists have generally responded to interaction complexity by simplifying the description of what constitutes the system, deliberately excluding potentially interacting members of the epidemiological network. While this approach has produced some successes, particularly in understanding specialist pathogens with simple transmission cycles and limited numbers of interacting hosts and vectors, it also carries some potentially serious weaknesses in both theoretical and applied realms (as highlighted by analyses of the boundary specification problem in network analysis; e.g. [11]).

An alternative approach to system simplification (i.e. rather than selectively picking out a small subset of interacting species to consider as “the system”) comes from the field of community ecology, in which researchers have attempted for decades to deconstruct the complexity of food webs [12]. Concepts such as trophic levels and foraging guilds have played an important role in the development of ecological theory; and Elton’s trophic pyramid, in which differences in the biomass of different trophic levels are explained by the second law of thermodynamics, is one of community ecology’s most fundamental generalisations. Many of the approaches that have been developed for food web analysis in ecology are readily applicable to the analysis of the ecology of pathogen transmission in multi-host systems [1,2,13,14].

The idea underlying functional group analysis is that broad, community-level trends in processes of interest can be detected by replacing a taxonomic classification of hosts with a classification that groups hosts according to their functional role in the epidemiology of a pathogen or a group of pathogens [15]. Although “AIV” describes a group of pathogens, we treat it as a single pathogen, ignoring AIV subtype variability, because the sparse information available does not suggest that AIV modes of transmission significantly differ among subtypes (see [16,17] for details). Hosts in an epidemiological functional group (EFG) share a common function in the epidemiology of the pathogen(s) of interest. We used the concept of EFGs to (1) investigate the ecology of AIVs in three different wild bird communities in southern Africa; and (2) critique the current scientific paradigm for field investigations of AIV in wild birds.

We used a wild bird census dataset to first allocate species to EFGs according to two epidemiological functions (reservoir vs. non-reservoir species, and the potential to introduce AIV strains through migratory behaviour; note that the functional groups, as described later, differ from the functions themselves) according to current AIV epidemiology dogma. We ranked each group in relation to their expected contribution to pathogen prevalence and then used our rankings to calculate relative *a priori* risk for each group. We then compared these *a priori* relative risks (which are effectively predictions, generated by accepted knowledge) to our empirical data on observed prevalence per group. In other words, we used EFGs as a way of exploring the degree to which empirical data match commonly held assumptions, rather than adopting the commoner approach of attempting to classify species into EFGs based on our own data. Our results suggest that commonly held assumptions may require some re-thinking.

## Materials and methods

### Study sites

We undertook research on bird communities at three geographically distinct sites in southern Africa. (1) Barberspan Nature Reserve (BAR) in the North West Province of South Africa, is a RAMSAR wetland of total area varying between 1000 and 1700 ha (GPS coordinates 26°35’00”S, 25°35’30”E); (2) Strandfontein wastewater treatment works (STR) in the Western Cape Province, South Africa, is a 319 ha water body located near Muizenberg on the immediate periphery of the city of Cape Town (GPS coordinates 35°05’00”S, 18°30’45”E); and (3) the Manyame-Chivero dams (MAN) in Zimbabwe, which are man-made impoundments that are linked by the Manyame river and were built in the 1950s to supply the city of Harare with water (GPS coordinates 30°30’30”, 17°45’00”). They cover areas of 6500 and 18 500 ha respectively. More information on the sites is available as supplementary material in Cumming et al. [17]. We selected our study sites based on three main criteria: (1) their designations as Important Bird Areas (as recognised by Wetlands International) with a high ornithological diversity; (2) the location of sites along a latitudinal gradient; and (3) their feasibility as study sites, which in this case meant finding a good compromise between the first two criteria, the need to sample each site at a high frequency, and the constraints imposed by available financial and human resources.

### Baseline data

All necessary permits to undertake this study were obtained from the relevant authorities; at STR, from the Cape Nature and Cape Town City Council; at BAR, from the North Wets Parks and Tourist Board; at MAN from the Governmental Veterinary Services and the Park and Wildlife Management Authority from Zimbabwe.

Bird census data were collected, using standardised point counts, from February 2007 to May 2009. Each point count consisted of a 10-min habituation period followed by a 30 min focal count of all birds in a semi-circle of 150m radius, facing the waterbody. Point counts were undertaken at 12 to 15 points at each of our three sites (BAR, STR and MAN) and were repeated four times at each location over five days during each counting and sampling session. Sessions were repeated every two months [17].

The prevalence of AIV (“estimated prevalence”) was estimated by capturing and sampling birds at each site every two months, over the two-year period from February 2007 to March 2009. Capture sessions were undertaken during a week-long intensive sampling period immediately after each 5-day counting session. Wild birds were caught using walk-in traps, mist nets and with occasional use of spring- or cannon-nets placed near the water’s edge. Diagnostics were performed using a real-time reverse transcription PCR technique on cloacal and tracheal swabs stored in a viral transport medium (Hank’s salt solution with antibiotics and fungicides) and kept in liquid nitrogen containers before delivery at the laboratory. Additional details on the protocols have been published in Cumming et al. [17].

Initially, the sampling protocol implemented in the three study sites was designed according to current knowledge about AIV epidemiology in wild birds. As almost no information was available from southern African ecosystems on AIV, it was decided to test the assumption that anseriforms and to a lesser extent charadriiforms were the main reservoirs of AIV in southern Africa. Capture methods were therefore chosen to maximize duck captures. All “by-catch” species were also sampled for AIV.

### Data analysis

Our analysis followed four main steps: (1) allocating bird species to EFGs, based on known characteristics of AIV ecology in wild birds, and assessing semi-quantitatively the risk associated with each EFG; (2) comparison of the waterfowl communities’ characteristics across the 3 sites by using biodiversity indices; (3) comparison of how representative the bird sampling was of the observed avifauna at each site; and (4), integrating the information gathered through the comparison of waterfowl communities, comparison between wild bird communities and composition of the bird captured sample and the estimated prevalence for each EFG in each study site to explore the relevance of the current AIV in wild bird dogma in these three ecosystems. Although we did obtain time series of prevalence, the temporal data exhibit high levels of variation and we have not included them in this manuscript for the sake of clarity (see [16,17] for detailed analyses).

#### Allocating bird species to EFGs

In order to reduce the complexity of the multi-host systems studied (several hundred bird species in each ecosystem), we defined two epidemiological functions (EF), with relevance to AIV epidemiology, on the basis of which we could allocate host species to EFGs. Hosts can play a limited number of roles (e.g., reservoir, dead-end host) in the epidemiology of a pathogen. Epidemiological functions related to each role can thus be used to regroup hosts into EFGs. Our initial assumption was that host species belonging to the same EFG would share more AIV epidemiology-related traits than host species in other EFGs. The two epidemiological functions (EFs) that we considered were (1) the maintenance and (2) the introduction of AIV strains. Note that for each epidemiological function, several different epidemiological functional groups exist. Both EFs reflect current and mainstream understanding of AIV ecology in wild birds.

The first EF relates to the AIV maintenance capacity of different bird species. The target population (according to the definition of [2]) is at risk of AIV transmission from the maintenance population either directly or indirectly through the non-maintenance population. The anseriforms and charadriiforms are bird orders that are considered globally to be reservoirs for AIV, and many studies consider only these two orders for epidemiological investigations (e.g. [18,19]). If there is an endemic AIV cycle in southern Africa, we hypothesized that anseriforms and potentially charadriiforms would constitute the maintenance community. We allocated anseriforms and charadriiforms into two different EFGs because they usually do not share either the same AIV prevalence or the same subtype pool, and do not always share transmission pathways [7]. In Africa, a role as a reservoir for both groups has been suggested by recent studies [16,17,20,21]. The other bird orders have not been sufficiently investigated to assign them different roles in viral maintenance. According to currently accepted assumptions about AIV ecology in wild birds, non-anseriform and non-charadriiform species are not associated with particular roles in AIV epidemiology. We thus grouped all of these species into the same EFG. This resulted in three epidemiological functional groups for AIV maintenance: *Ans* (anseriforms), *Cha* (charadriiforms) and *RoC* group (Rest of Community), with the latter category containing all non-anseriform and non-charadriiform bird species. If anseriforms and charadriiforms represent the main reservoir of AIV in southern Africa, the *RoC* group should play a minor role in the ecology of AIV, with occasional spill-over of AIV strains triggering infections, and the estimated prevalence in this group should thus be lower than in the two other groups across the study. Anseriforms usually present a higher AIV prevalence than charadriiforms [7]. Based on this information, relative risks of 3, 2 and 1 were allocated to *Ans*, *Char* and *RoC* respectively. Values allocated here are semi-quantitative and should be considered simply as ranking the expected prevalence for each group, rather than as describing its relative magnitude.

The second EF concerns the potential of bird species to introduce AIV strains into the ecosystem from different ecosystems across regions or continents. As birds move or migrate away from a given ecosystem, they will be exposed to a greater variety of AIV strains and could introduce those strains into the ecosystem on their return. The role of migrating wildlife in the spread of diseases has been recently reviewed [22]. Although long-distance migration is not systematically correlated with pathogen dispersal, the role of wild birds in spreading LPAI has been documented [23,24]. Depending on the circulation of AIV in the ecosystem under study, the introduction of exogenous strains could trigger epizootics if no cross-immunity against these strains exists. Such introductions could also play a role in reassortment processes and the emergence of new strains [25,26]. We thus allocated birds in our study communities to the following epidemiological functional groups relating to pathogen introduction: (a) Long range spreader or Palaearctic (*Pal*) migrant, migrating from Eurasia where a higher prevalence of AIV can occur at some times of year [27]; (b) Middle range spreader or Afrotropical (*Afr*) migrant, migrating North of the equator in Africa; (c) Local spreader or nomad (*Afr*, see discussion below), moving regionally to follow resources and/or undertake moult or breeding-related local migrations; and (d) Non spreader or Resident (*Res*) bird with limited local movements.

Despite the availability of detailed information about wild bird ecology in southern Africa [28], the movement ecology of many species remains unclear, particularly when geographically distinct populations of the same species behave differently (e.g., [29]). We therefore decided to group medium and local-scale spreader species into a single *Afr* (mobile Afro-tropical) group. A role for Palaearctic birds in the introduction of Eurasian AIV strains in Africa has been suggested [30,31]. If there is no endemicity of AIV in southern Africa, we hypothesized that Palaearctic migrants should introduce AIV regularly in these ecosystems. By contrast, a community dominated by the “Resident” EFGs should experience little AIV circulation. Based on these assumptions, relative risks of 3, 2 and 1 were allocated to *Pal*, *Afr* and *Res* respectively.

We then combined both EFs by creating a matrix of 3×3 EFGs as used for the analyses. The relative risk at the EFG level was calculated by multiplying the relative risks of each EF (Table 1), providing a ranking of AIV prevalence between groups to be challenged by field data. Risk values were multiplied, as is the norm for probabilistic estimates of risk, because group scores captured the non-linear interactions between EF1 and EF2 risks.

**Table 1:** Epidemiological functional groups and relative risk

**EF2**	**EF1**	**Anseriforms*Ans = 3***	**Charadriiforms*Cha = 2***	**Rest of Community*RoC = 1***
Resident *Res = 1*		*Ans-Res = 3*	*Cha-Res = 2*	*RoC-Res = 1*
Afro-tropical migrant *Afr = 2*		*Ans-Afr = 6*	*Cha-Afr = 4*	*RoC-Afr = 2*
Palaearctic migrant *Pal = 3*		*Ans-Pal = 9*	*Cha-Pal = 6*	*RoC-Pal = 3*

Epidemiological functional groups used in this study, based on the two epidemiological functions related to the maintenance and introduction potential respectively of AIV in Southern African ecosystems. Numbers represent qualitative estimations of the AIV relative risk for each epidemiological function and for each EFG. For each cell, the qualitative estimation of the relative risk is calculated by multiplying values of the relative risk of EFGs from EF1 and EF2.

Chi-square tests were used to compare prevalence between sites and EFGs. Spearman Rank Correlation tests were performed to compare proportion of observed vs. captured EFGs and semi-quantitative variables of risk estimation.

#### Comparison of bird communities between sites and bird sampled vs. counted

Two indices were calculated to describe the waterfowl community in the three sites: species richness (total number of species) and Shannon’s diversity index [32], which combines species richness and abundance. The influences of both host richness and abundance have been shown to be important for epidemiological dynamics [33]. Both indices were calculated across the two years of count data. The bird species of the 3 sites were allocated to the 9 EFGs using available regional knowledge [28] and the composition of these groups was compared across ecosystems (see below). The relative bird density was represented by the total number of birds observed divided by the total number of counts for that site; no additional correction for area was necessary because all counts were undertaken within a semicircle of 150m radius.

We estimated how well our sampling represented the observed bird community and the bias induced by the bird capture techniques and the “catchability” of waterfowl species by comparing the proportions of each bird group captured and observed across the two years of capture. A Spearman rank correlation test was performed between observed and captured birds in each of the three sites in order to assess how representative the sample composition was *vis a vis* the observed bird community.

#### Prevalence and AIV risk estimation for EFGs

For each EFG and for each site, we calculated the estimated prevalence by dividing the number of positive birds by the number of birds sampled. We also estimated the “*a priori* risk” of AIV by multiplying the relative risk presented in Table 1 by the EFG proportions observed (from the total counts) in each different ecosystem. We assumed a similar weight for both maintenance and introduction functions. These “*a priori* risk” values provide semi-quantitative predictions about AIV circulation in each of the EFGs according to current knowledge of AIV in wild birds.

We then calculated an “estimated risk” by multiplying the AIV prevalence calculated at the EFG and study site level by the EFG proportions observed (again from the total counts) in each of the ecosystems. The “*a priori* risk”, which captures current knowledge about AIV epidemiology, was then compared to the “estimated risk”, which captures observed prevalence and community composition in our three sites in southern Africa. For both risks, we summed the risk value across EFGs to calculate a “Site community” value, which is an estimation of both risks at the study site level. We compared the “*a priori* risk” with the “estimated risk”, excluding the Resident and Palaearctic anseriforms that were absent from all three ecosystems.

## Results

### Comparison of waterfowl communities observed and captured between sites

**Figure 1:**
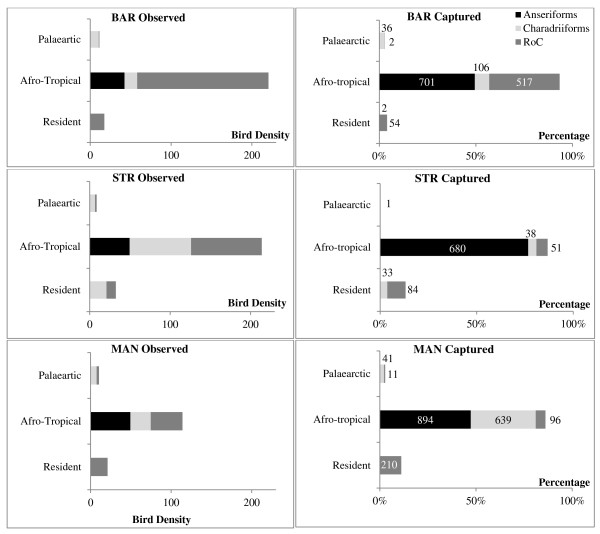
**Community observed and captured in the three sites.** Community observed (left) and captured (right) in the three sites according to EF 1 & 2 groups. Bird density (“Observed”) is calculated by the number of birds observed divided by the number of counts (counts implemented in a given area). Bird abundance (“Captured”) is the pecentage of birds captured (numbers indicate the number of birds captured per EFG). Dark grey = anseriforms, Medium grey = RoC and Light Grey = charadriiforms.

The bird community at MAN had higher species richness, a higher Shannon index and a lower relative bird density than STR and BAR (Table 2). STR was less diverse (139 against 199 species recorded) and the values of the Shannon index were smaller than in BAR. The avian community composition relative to EFGs across the three sites differed (Table 2). BAR and STR were dominated by *Roc-Afr*, MAN by *Ans-Afr.* In all 3 ecosystems, *Ans-Afr* and *Roc-Afr* represented more than 17% of the birds observed and there were no *Ans-Res* and *Ans-Pal* and only a few *Roc-Pal*. Few *Cha-Pal* (between 2.6 and 5.1% of all counted birds) were present in these ecosystems. MAN, STR and BAR did not differ in anseriforms (mainly Afro-tropical migrants) density, but did differ in the relative abundance of anseriforms in the total community (Table 2, Figure 1).

**Table 2:** Indicators of waterfowl community diversity

		**BAR**	**STR**	**MAN**
	**Bird Obs/Count**	246 ± 537	234 ± 216	144 ± 171
	**Species richness**	198	138	249
	**Shannon’s index**	2.72	2.95	3.54
**EFG**	***Ans-Res***	0.0%	0.0%	0.0%
	***Ans-Afr***	**17.0%**	**19.4%**	**34.0%**
	***Ans-Pal***	0.0%	0.0%	0.0%
	***Cha-Res***	0.2%	8.2%	0.1%
	***Cha-Afr***	6.3%	**30.0%**	**17.3%**
	***Cha-Pal***	4.3%	2.6%	5.1%
	***RoC-Res***	6.8%	4.5%	**14.4%**
	***RoC-Afr***	**65.1%**	**34.6%**	**27.1%**
	***RoC-Pal***	0.3%	0.7%	2.0%
	**Total**	100.0%	100.0%	100.0%

“Birds Obs/Count”: average number of birds observed per count and standard error displayed; “Species richness”: number of species observed across the two years; “Shannon’s index” diversity index. Proportions of each combined epidemiological functional group (EFG) are displayed in each ecosystem (Ans = anseriforms, Cha = charadriiforms, RoC = Rest of Community, Res = resident, Afr = afro-tropical, Pal = palaearctic). In bold, dominant EFGs for each ecosystem.

In all three sites, the *Ans-Afr* EFG was over-represented in the sampled birds (Figure 1), reflecting the objectives of the sampling protocol. In addition, some bird families that were abundant in the counts were poorly represented or absent from the captured birds: the *RoC-Afr* group for all three sites and *Char-Afr* in STR. No *Cha-Pal* were captured at STR. Only in BAR, the captured and observed EFGs were correlated (STR: Spearman’s r = 0.37, *p* = 0.497), BAR: Spearman’s r = 0.92, *p* < 0.010, MAN Spearman’s r = 0.83, *p* = 0.058).

### AIV prevalence and risk comparison for EFGs

**Figure 2:**
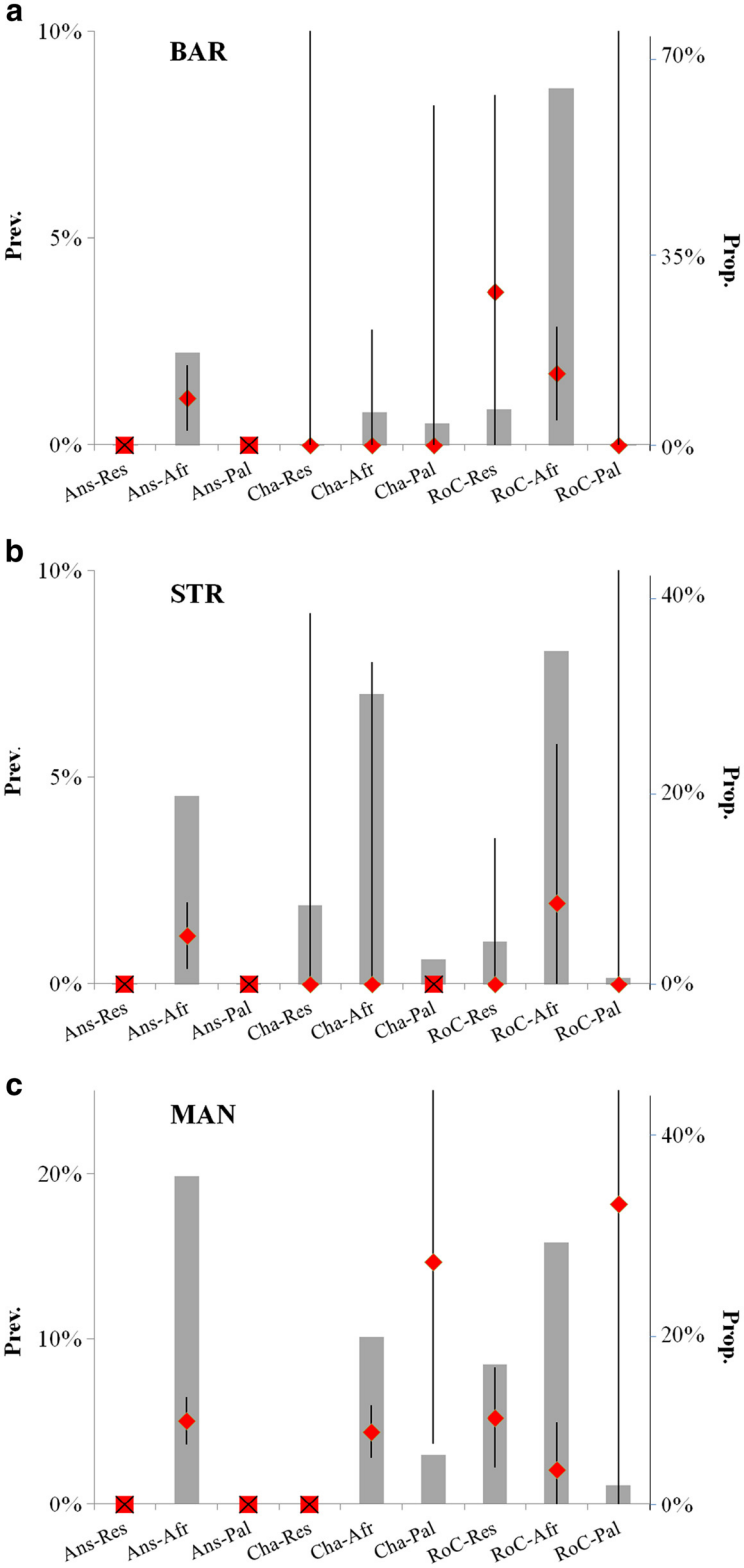
**AIV Prevalence for each EFG in relation to bird community composition in the three sites.** For each site (BAR, STR, MAN): **a**) diamonds represent AIV prevalence with 95% confidence interval (left axis) for each combination between EF1 & EF2 (Ans = anseriforms, Cha = charadriiforms, RoC = Rest of Community, Res = resident, Afr = afro-tropical, Pal = Palaearctic migrant); **b**) grey bars represent proportion of each bird group in the bird community observed (or counted) during the 2 years of the project (right axis).

The anseriforms afro-tropical group represented the only anseriforms present in all three sites and their AIV prevalence was 1.1, 1.2, and 5.0% respectively for BAR, STR & MAN (95% Confidence Intervals being [0.7:1.9], [0.4:1.7], [4:5.9] respectively) differing significantly between MAN and both BAR and STR (both chi-square tests being highly significant, *p* < 0.001). *Cha-Res*, *Cha-Afr* and *Cha-Pal* had zero prevalence at both BAR and STR, albeit with small sample sizes (maximum possible prevalence 98.0%, 2.8%, 8.2% for BAR and 9.0%, 7.8% and NA for STR respectively for the 3 EFGs at 95% Confidence Interval). At MAN, *Char-Afr* had a relatively high AIV prevalence (as for *Char-Pal*) but with a large confidence interval (Figure 2). The *RoC-Afr* group had detectable prevalence in the three sites, *RoC-Res* for BAR and MAN and *Roc-Pal* had a high prevalence in MAN but with a small sample size. Any bird groups representing more than 15% of the community had detectable prevalence of AIV in all three sites with the exception of *Cha-Afr* in STR (but with only 38 individuals sampled).

All groups present in MAN had a detectable mean prevalence. The MAN prevalence for *RoC-Res* and *RoC-Afr* were significantly higher than BAR *RoC-Afr* (chi-square test, *p* < 0.01 and *p* < 0.05 respectively) and higher but not significantly different from BAR *RoC-Res* and STR *RoC-Afr* (also the sample size of this group was small). The prevalence of the *RoC-Afr* and *Cha-Afr* groups in MAN were not significantly different from the *Ans-Afr* group. In BAR and STR, AIV prevalence in well-sampled groups appeared similar.

According to “*a priori* risk” estimation based on current AIV in wild bird knowledge and the bird community composition, we expected AIV site prevalence to be higher in MAN, followed by STR and lastly BAR (“Site community” row in Table 3). The “estimated risk” using field prevalence and bird community composition also predicted a difference between the sites, with MAN having the higher risk estimation, followed by BAR and STR. This site level approach can be compared with an alternative hypothesis, based on current understanding of AIV epidemiology in wild birds. Without taking into account the bird community composition, we would expect a similar AIV prevalence in the three ecosystems because the density of anseriforms was similar in the three ecosystems (Figure 1, *Ans-Afr* = 42.5, 49.5 and 49.5 birds per counts/unit area for BAR, STR and MAN respectively). None of “*a priori* risk” and “estimated risk” were correlated (Table 3, for BAR, STR and MAN respectively, Spearman’s r = 0.388, *p* = 0.396; Spearman’s r = 0.449, *p* = 0.302; Spearman’s r = 0.609, *p* = 0.167) indicating that *a priori* assumptions about the epidemiological role of the host community did not fit our field data.

**Table 3:** Sample size, estimated prevalence and relative risk for each epidemiological functional group

	**BAR**			**STR**			**MAN**		
	***n***	**Estimated Risk**	***A priori*Risk**	***n***	**Estimated Risk**	***A priori*Risk**	***n***	**Estimated Risk**	***A priori*Risk**
***Site community***	**1418**	**1.58**	**2.85**	***887***	**0.91**	**3.40**	***1891***	**4.9**	**3.64**
***Ans-Res***	0	na	0.00	*0*	na	0.00	*0*	na	0,00
***Ans-Afr***	701	0.19	**1.02**	*680*	0.23	**1.16**	*894*	**1.71**	**2.04**
***Ans-Pal***	0	na	0.00	*0*	na	0.00	*0*	na	0.00
***Cha-Res***	2	0.00	00.00	*33*	0.00	0.16	*0*	na	0.00
***Cha-Afr***	106	0.00	0.25	*38*	*0.00*	**1.20**	*639*	0.76	0.69
***Cha-Pal***	36	0.00	0.26	*0*	na	0.16	*41*	*0.75*	0.31
***RoC-Res***	54	*0.25*	0.00	*84*	0.00	0.00	*210*	0.76	0.00
***RoC-Afr***	517	**1.13**	**1.30**	*51*	**0.68**	0.69	*96*	0.56	0.54
***RoC-Pal***	2	0.00	0.01	*1*	0.00	0.02	*11*	*0.36*	0.06

Sample size, estimated risk and *a priori* risk for each epidemiological functional group in each of the three study sites. “Estimated risk” is the product of AIV prevalence calculated for each site across the 12 sampling sessions at the community level (row “Site community”) and for each EFG and of the proportion of the EFG in the bird community (Table 2); *n* = number of birds sampled; based on results presented in Cumming et al. [17]; “na” indicates that no birds of this group were sampled. “A priori risk” for each EFG was globally calculated by multiplying the relative risk for each functional groups (Table 1) and the proportion of each group in the bird community (Table 2). Values in italic indicate groups that would require more sampling because of relatively high “a priori risk” or high “estimated risk” combined with small sample size. Values in bold for EFG indicate the highest respective values when sample size is adequate for both “estimated risk” and “a priori risk”.

## Discussion

Analysing this epidemiological dataset in accordance with the current dogma of AIV in wild birds, anseriforms prevalence appears to drive prevalence at the community level in each ecosystem (Table 3) [16,17]. By including bird community data and the composition of the captured sample, and taking into account the EFG approach, we obtain a different perspective: (1) different bird communities predict different AIV risks (“*a priori* risk”) using the EFG approach in the three ecosystems, a result validated by the “estimated risk” using field AIV prevalence; (2) sampling bias can explain discrepancies between the “*a priori* risk” and “estimated risk” for AIV prevalence; and (3) anseriforms play an important role in AIV epidemiology in waterfowl in the three ecosystems, as assumed by the current understanding of AIV ecology in wild birds, but other bird groups identified at the EFG level show unexpectedly high prevalence, and could play a role in the local epidemiology of AIV.

The EFG approach thus appears to be successful in improving our understanding of the role of wild birds in the epidemiology of AIV by highlighting potential epidemiological functions for unconsidered bird groups, identifying gaps in knowledge or sampling (see in Table 3, EFGs highlighted in italic) and suggesting new hypotheses. The EFG approach also carries the benefit of making better use of “by-catch” samples, which are often portrayed in AIV studies as secondary-level data [19].

We expected that different host community compositions in different ecosystems would lead to different epidemiological patterns. Our data show that bird communities differed substantially between sites and, as a consequence, the site “*a priori* risk” related to the two epidemiological functions varied (Tables 2 and 3). The larger size of the MAN ecosystem compared to the two other wetlands, and its more tropical location, may explain many of the observed differences in density and species richness. Some similarities in the bird communities were nonetheless observed across the sites. For instance, most anseriforms in southern Africa are afro-tropical species, as few Palaearctic anseriforms reach southern Africa. Other Palaearctic migrants (e.g. ruffs *Philomachus pugnax* and common sandpipers *Actitis hypoleucos*) arrive in the region from Eurasia in late September and early October, but most are present in relatively low numbers (Figure 1). The “*a priori* risk” of AIV introduction through intercontinental migration is therefore low compared to the same risk through intra-continental movements as most of the birds at our study sites remain within the afro-tropical region (88.4, 84.0 and 78.4% for respectively BAR, STR and MAN). The avian community composition at MAN appeared to be more favourable to AIV maintenance because it is dominated by afro-tropical anseriforms. At STR, the large presence of afro-tropical charadriiforms also suggested the possibility of AIV maintenance. At BAR, the *RoC* group dominated and we expected little AIV circulation (Table 3).

Our field data for AIV prevalence partially supported the “*a priori* risk” estimation based on current AIV ecology dogma. However, they did not support the hypothesis of equivalent AIV prevalence in the three ecosystems, despite similar densities of anseriforms. This suggests that sampling anseriforms without taking into account the rest of the bird community can lead to false conclusions. MAN has a higher AIV prevalence at the community level compared to BAR and STR, as indicated in Figure 2. However, the prediction that STR would have a higher “*a priori* risk” than BAR (Table 3) was not corroborated by observed “estimated risk”. Our principal explanation for this discrepancy is the bias in the captured sample compared to the bird community composition at STR (Spearman rank correlation test). STR is the only site with a non-significant correlation coefficient between observed and captured bird community composition. We would expect that increased sampling in abundant EFGs such as afro-tropical charadriiforms and *RoC* groups at STR would increase our estimate of its site AIV prevalence and its “estimated risk”. At BAR and MAN, the contribution of each EFG to the AIV prevalence is in agreement with field predictions in EFGs with a large sample size (i.e. more than 100 birds sampled).

Current knowledge about global AIV epidemiology applies to some extent to AIV epidemiology in these three southern African ecosystems. Notably, the important role of afro-tropical anseriforms in the epidemiology of AIV is confirmed in southern Africa [16,17]. However, higher than predicted AIV prevalence in other bird groups challenges the hegemony of anseriforms as the primary actor in the maintenance of AIV in these ecosystems. Firstly, the AIV prevalence estimated in the resident *RoC* group in BAR and MAN is not significantly different from the prevalence in the afro-tropical anseriforms group across the two years of the study. The same observation can be made for afro-tropical charadriiforms and to a lesser extent for the Palaearctic *RoC* group (with a high prevalence but a small sample size leading to a high maximum undetectable prevalence) at MAN. For all three ecosystems, the majority of AIV infected birds (estimated by multiplying the prevalence by the proportion of the group in the community in Figure 2) would not belong to the afro-tropical anseriforms group, contrary to what might be expected for the reservoir of the disease. In BAR and STR, for example, the majority of the infected birds would belong to the afro-tropical *RoC* group. Finally, all EFGs but one that represent more than 15% of the community have a detectable prevalence, suggesting a frequency-dependent role of EFGs in relation to AIV prevalence. A role for these EFGs in the persistence or maintenance of AIV in our study ecosystems cannot be ruled out, even though this conclusion would go against current dogma surrounding AIV epidemiology in wild birds [34]. Most studies of AIV ecology in wild birds have been implemented in temperate climates [7]. The current dogma in this field therefore comes from studies implemented in specific biophysical conditions (e.g. climatic condition, ornithological diversity) that have been shown to influence the epidemiology of AIV [35]. Often, this dogma is taken for granted when studies are implemented in other regions with different biophysical conditions. Our results indicate that more attention should be given to designing local hypotheses in reference to global assumptions: some space should be left for alternative hypotheses and the inclusion of count data and the sampling of other bird species than anseriforms and charadriiforms will serve this purpose.

The resident *RoC* groups represent more than 100 species in each ecosystem. Most of the species in these groups had no positive sample and a small sample size. A few species drive prevalence estimates at the group level but lack an adequate sample size. Cumming et al. [17] identified some of the families or species that are high priorities for further sampling: for example, Hirundidae (1 positive out of 8, 7.7%), Alaudidae (3 out 24, 12.5%), and Motacillidae (2 out of 43, 4.7%). For some terrestrial passerine species, experimental data suggest a potential role in virus shedding (e.g. [36-39]). Too few samples from Palaearctic species were obtained through this study to provide a clear picture of their role (*n* = 2, 1, and 44 respectively for BAR, STR and MAN with only 8 positives in MAN). However, the 17% prevalence estimated for Palaearctic charadriiforms in MAN (*n* = 35) indicates the need for more information about this group.

This study was implemented to provide the first longitudinal AIV information for these ecosystems. Its initial design was similar to most wild bird AIV surveys, focusing primarily on anseriforms and charadriiforms that were assumed to play an important role in AIV epidemiology in all ecosystems. The sampling of all birds captured during the protocols allowed us to investigate additional hypotheses about AIV ecology in wild birds. What is more, by adding bird count data, we were able to combine our sampling and prevalence data with available ornithological knowledge to allocate bird species into EFGs and thus to simplify the multi-host complexity of the study system. Our data support the idea that some EFGs play an important role in the persistence and/or maintenance of AIV in southern African ecosystem. They also imply that comparisons of prevalence data from multiple sites (even if the sampling was done at similar time) are compromised if environmental and ecological variability is not accounted for. To understand key issues such as HPAI strain emergence and local maintenance of AIV, the role of the avian community as a whole must be considered; selective sampling of a largely unjustified subset of species from within an extensive interacting host community can no longer be justified. In general, our analysis provides strong support for the argument that functional approaches to complex host-pathogen systems can offer valuable ways of reducing the complexity of interactions to a more manageable level.

## Competing interests

The authors declare that they have no competing interests.

## Authors’ contributions

AC contributed to conception and design of the study, data collection in Zimbabwe and provided the first and subsequent drafts of the manuscript. MdGW contributed to conception and design of the study and commented substantially on several drafts of the manuscript. MN contributed to conception and design of the study, data collection in South Africa and commented substantially on several drafts of the manuscript. GSC contributed to conception and design of the study, data collection in South Africa and commented substantially on several drafts of the manuscript. All authors read and approved the final manuscript.
